# State of the Art Physiotherapist-Led Approaches to Safe Aging in Place

**DOI:** 10.1186/s40945-022-00142-5

**Published:** 2022-08-01

**Authors:** Christopher M. Wilson, Sara K. Arena, Lori E. Boright

**Affiliations:** Physical Therapy Program, Oakland University, 433 Meadow Brook Dr, Rochester, MI USA

**Keywords:** Geriatrics, Comprehensive geriatric assessment, Falls, Independent living, Prevention, Safety, Home modification, Balance, Exercise

## Abstract

**Introduction:**

Safe aging in place (SAIP) is when an older adult can successfully and comfortably remain in their home despite increasing barriers, including falls. Various physical, medical, psychological, and psychosocial factors may individually or cumulatively impact an older adult’s ability to safely age in place. Physiotherapists should assess not only items traditionally considered within their scope of practice but should select efficient and effective outcome measures to quantify other domains of health. A comprehensive geriatric assessment (CGA) is an evidence-based clinical assessment which identifies medical, psychosocial, and functional limitations of an older person. The CGA is useful to dictate individualized exercise/intervention prescription to address identified areas of increased risk.

**Purpose and importance to practice:**

The purpose of this Masterclass is to describe key screening, assessments, and interventions to facilitate SAIP and to provide overviews of currently available programming and care delivery models applicable to physiotherapist practice.

There are a wide variety of outcome measures and interventions that vary in depth, validity, and reliability. Measures selected for inclusion in this Masterclass were chosen based upon their clinical utility with respect to time and resource constraints and ease of administration during a comprehensive assessment for SAIP in community-dwelling older adults. Measures recommended for assessing physical function were the Short Physical Performance Battery, the Timed-Up-and-Go, the 30 second chair rise test, and the Four Test Balance Scale. Additionally, measures from the heath domain (e.g., Functional Comorbidity Index) and the environmental domain (e.g., Home FAST) are recommended. Relative to interventions, the Otago Exercise Program, motivational interviewing, home modifications, and leveraging technology are recommended. Partnerships with community-facing organizations facilitate utilization of resources for sustainable SAIP. The Home-based Older Person Upstreaming Prevention Physical Therapy (HOP-UP-PT) program is one approach led by physiotherapists framed in the screening, assessments, and interventions discussed in this Masterclass with strong scientific grounding.

**Conclusion:**

Programs integrating both community and healthcare approaches have the strongest evidence for their utility; however, implementation for these preventative approaches are lagging behind the increased need due to the substantial population growth of those over 65 years.

## Background

Although many older adults strive to remain safely in their homes, often termed “safe aging in place” (SAIP), this goal often becomes less feasible over time and may necessitate assistance to remain living at home safely. One major barrier to SAIP is fall risk. The cost of falls and the associated emergency room visits, hospitalizations, and nursing home care among older adults has contributed to skyrocketing stress on the healthcare system globally [1, 2]. Research suggests that approximately one-third of individuals 65 years and older will fall annually with the physical outcomes of each fall event ranging in severity from no injury to death [3]. Furthermore, increased fear of falling and decreased confidence when performing activities of daily living may increase future fall risk [4, 5]. The alarming rate of falls among older adults, in combination with reports that 38% of these falls will require medical treatment, warrants proactive measures to reduce falls and fall risk in the aging population [6]. In addition to the personal burden of falling, healthcare expenditures associated with emergency department visits after a fall averages $3038 United States Dollars (USD) and increases to $38,412 USD if an individual requires hospitalization [7]. Therefore, even a moderate reduction in falls can improve an older adult’s ability to safely age in place and has the potential for substantial cost savings [8]. To augment an older adult’s ability and resources to safely age in place, key physical, medical, and social determinants of health must be addressed.

Rowe and Kahn defined successful aging to include a low level of disability, having high cognitive and functional capacity, and actively engaging in life events [9]. This definition was updated in 2015 to include influences on successful aging that encompass the person’s interpersonal environment. These include social or family relationships, support networks, community accessibility, and availability of community programming [10]. The term successful aging, most frequently utilized in the United States, and active aging, a phrase more common in Europe, both represent a similar concept. Active aging is describe as having a “holistic approach including quality of life, mental and physical well-being, and social participation” [11]. The concept of active aging also encompasses development of supportive policies and societal responsibilities, in addition to facilitating individual responsibility for active aging [11].

**Fig. 1:**
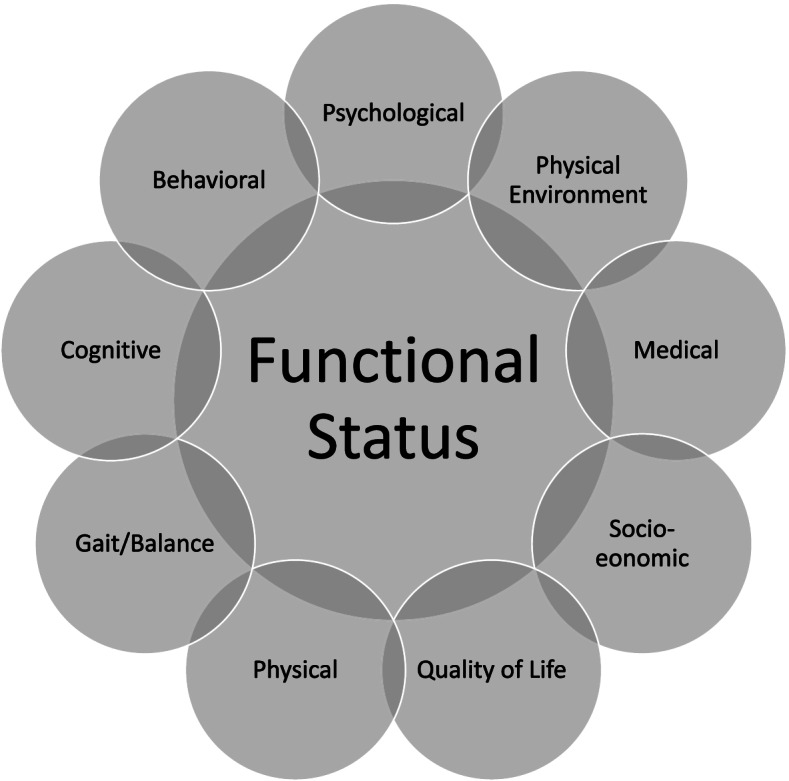
Comprehensive Geriatric Assessment Domains

One of the cornerstones of modern geriatric care is the comprehensive geriatric assessment (CGA). A CGA is a multifactorial assessment process ultimately leading to individualized interventions. The CGA was developed to mitigate high rates of institutionalization in the frail older population and to address readily recognizable problems among these individuals [12]. Although the procedures described in this manuscript are intended for a physiotherapist-led comprehensive assessment for SAIP in community-dwelling older adults, the domains commonly assessed in an interdisciplinary CGA were utilized due to their wide acceptance and scientific body of evidence. Figure 1 highlights the wide variety of domains that should be quantifiably assessed. If there is an area of concern or deficit in one of the domains, the provider should either provide direct interventions or consider referral to an interdisciplinary team member. Studies have repeatedly shown that social, community, and environmental factors can have a substantial impact on the ability of older adults to thrive and live independently, therefore these factors must be addressed [12].

The purpose of this Masterclass is 1) to describe individualized approaches to screening, assessments, and interventions of older adults aimed at SAIP and reducing fall risk, and 2) to provide overviews of programming and care delivery models already available within the existing literature that support SAIP initiatives including an in-depth description of one physiotherapist-led program that utilizes this approach.

## Assesment

Various physical, medical, psychological, and psychosocial factors may all individually or cumulatively impact an older adult’s ability to age successfully and actively. Therefore, physiotherapists should assess not only the items considered within their traditional scope of practice, (e.g., strength, balance, functional mobility) but to also select efficient and effective outcome measures to quantify many of these ‘other’ domains of health. Due to the myriad issues that may cause difficulty with SAIP and active aging, a dilemma exists for physiotherapists to determine which domains to assess and how thoroughly; this is highly individualized based on each older adult’s personal circumstances. The intention of this Masterclass is to describe the most common and clinically relevant domains; however, there may be other domains that a physiotherapist may need to assess and address that are not highlighted in this article (e.g., vision, hearing, financial stability, food security, housing security). Although there is a plethora of valid and reliable outcome measures with ample breadth and granularity in each instrument, the authors have curated those measures with utility for clinical practice within the context of a CGA (Table 1). As there are numerous domains to be assessed in a CGA, a primary consideration for the authors when selecting measures to be described was the amount of time required to administer and ease of administration (e.g., limited amount of equipment and training required to perform) with a prerequisite being adequate psychometric properties. It is recognized there are additional comprehensive, valid, and reliable assessments that can (and should) be administered when there is ample time during a physiotherapist evaluation; however, these often require increased time, training, and equipment which increases the risk of the physiotherapist to not have time to assess other equally important domains. This may result in unacceptable gaps in a plan to comprehensively address SAIP.

**Table 1:** Evidence Based Measures and Estimated Time to Complete

Health Domain	Assessment Tools	Estimated Time to Complete	Number of Items/Tests
Comorbidity	Functional Comorbidity Index	4–13 min	18 items
	Charlson Comorbidity Index	4–13 min	19 items
Blood Pressure	Electronic blood pressure cuff or manual sphygmomanometer and stethoscope	2–5 min	1 assessment
Polypharmacy	Beers Criteria and STEADI questions	3–5 min	5 lists of nearly 100 medications
Cognition	Mini Mental State Exam	7–8 min	21 items
	Mini-Cog	3 mins	3 tasks
	Trail Making Test B	2–5 min	1 task
Depression	Geriatric Depression Scale	7–10 min	30 items
	Geriatric Depression Scale Short Form	5–7 min	15 items
	Patient Health Questionnaire-9	3–6 min	9 items
Environment	Home FAST-HP	30–45 min	25 items
	Home FAST-SR	15 mins	88 items
Health Behaviors and Readiness for Change	Physical Therapy Healthy Lifestyle Appraisal	10 mins	18 items
Frailty	Fried Frailty Index	5–10 min	3 items, 2 tasks
Physical Function	Short Physical Performance Battery (includes 3- or 4-m gait speed, 5 times sit to stand, Four Test Balance Scale)	5–10 min	3 tasks
	Timed-Up-and-Go	1 min	1 task
	30 second chair rise test	1 min	1 task
	Four stage balance scale	2 mins	3 tasks
Falls Efficacy	Falls Efficacy Scale – International	5–10 min	16 items
	Falls Efficacy Scale International – Short Form	4–5 min	7 items
	Modified Falls Efficacy Scale	5–10 min	14 items

*Min(s)* Minutes, *STEADI* Stopping Elderly Accidental Death and Injury, *HP* Health professional, *SP* Self-report

### Comorbidities

One of the key components to determining the need for services and risk of future health or functional issues is risk stratification. Although there is not necessarily a strong direct relationship between physical performance and the number of comorbidities that a person has, the risk of disability does increase with the number of concurrent diseases [13]. Two of the most cited measures are the Functional Comorbidity Index (FCI) and the Charlson Comorbidity Index (CCI). The FCI is a list of 18 common diagnoses specifically selected to quantify the overall burden of these diagnoses from a variety of body systems including cardiac, pulmonary, musculoskeletal, and neurologic [14]. Limitations to the original FCI include that the measure does not have a direct relationship with overall mortality and does not consider the extent or severity of a disease (e.g., if one disease is quite severe, it may have a similar functional impact as multiple less severe diseases.) There is, however, a weighted version of the FCI that considers the severity of disease but may take more time to administer and requires the judgement of the clinician to estimate disease severity [15, 16].

The CCI predicts the 10-year survival in individuals with multiple comorbidities. It is widely utilized in pre-operative screening to establish the risk of adverse surgical outcomes or death [17, 18]. The severity of the 19 comorbidities are weighted from one to six. Within the CCI, there are several conditions that are not included in the FCI, such as HIV/AIDS and integumentary issues such as wounds [16]. Although no comorbidity index or questionnaire would be able to assess the myriad diagnoses that a person could possibly have, these two indices can be used to identify which individuals may be at future risk of negative outcomes or require increased healthcare services to mitigate risk through physiotherapist interventions or referral to other relevant healthcare practitioners.

Notably, neither of these comorbidity indices incorporate hypertension (HTN) in the composite scoring. However, given the variety of serious health sequalae associated with HTN (e.g., cerebral vascular accident, coronary heart disease, renal disease), a physiotherapist’s inclusion of blood pressure (BP) measures in routine care is essential [19, 20]. This assertion is further supported by reports that nearly two thirds of adult patients under the care of physiotherapists present with elevated BP readings [21–23]. Recommendations for best practices in prevention, detection, evaluation, and management of elevated BP are available [24]. Additionally, a BP decision making algorithm may be useful to guide patient management [25].

### Polypharmacy/medication

Polypharmacy and high-risk medication use among older adults are two areas that are well known to increase the risk of falls, possibly leading to injury or death [26]. As physiotherapists routinely encounter older adults consuming a variety of medications that lead to risk, a review and reconciliation of the medication list is considered best practice [27]. As non-prescribing providers, physiotherapists will have varying familiarity and comfort with reviewing a medication list. Therefore, using available internet databases to determine if medications may result in complications is warranted. There are two potentially useful clinical tools that may assist physiotherapists in determining if a person is at risk of medication-related issues which suggest referral to a physician or pharmacist for further evaluation. These are the US Center for Disease Control and Prevention’s (CDC’s) Stopping Elderly Accidental Death and Injury (STEADI) Medications Linked to Falls resource guide [28], and the American Geriatrics Society’s (AGS’) Beers Criteria [29]. The Beers Criteria, most recently updated in 2019 by the American Geriatrics Society are available at www.GeriatricsCareOnline.org. More specifically, the STEADI questions emphasize the risk of medication-related falls while the Beers criteria also incorporates additional potential medication-related adverse effects for older adults. See Table 2 for key questions that are useful in screening for pharmacological risk.

**Table 2:** Screening questions for medications and polypharmacy

[ ] Reviewed medications, dosages	
[ ] The client is taking more than 4 recurrent medications	
[ ] The client is taking more than 9 recurrent medications	
[ ] The client is taking one or more psychotropic medication (i.e. hypnotics, antidepressants and benzodiazepines)	

### Cognition and depression

Altered mentation and mood disorders are common geriatric syndromes that can greatly affect physiotherapist care and an older adult’s ability to age actively and independently [30]. As preventative care is grounded in client self-efficacy and safety to properly perform prescribed physical activity regimens, it is imperative that the physiotherapist quickly yet comprehensively assess cognition and mood. Therefore, three cognition assessments (Mini Mental State Exam [MMSE], Mini-Cog, Trail Making Test [TMT]), and two depression screens will be described.

The MMSE is a questionnaire rated on a 30-point scale and demonstrates moderately high reliability [31]. It has 21 items, 11 different tests, and was primarily developed to help quantify decline in persons with Alzheimer’s dementia. A score of 23 or less may indicate dementia and warrants further screening. Due to its reliability, validity, and widespread use, it is often used as a criterion standard for other cognitive assessments [32].

The Mini-Cog is a brief assessment consisting of a clock drawing task followed by a three-word recall. Each word correctly recalled is scored one point and a correctly drawn clock is scored as two points. As a screen for dementia, a cutoff score of <3 has been clinically validated [33]. The reduced societal use of analog clocks may render this tool obsolete in the future.

The TMT has two components, Part A and Part B, and both can be completed independent of one another. Each test requires a participant to connect circles with consecutive letters or numbers within each circle. The test is timed, and total time includes correction if mistakes occur. Part B is more complex and requires a person to alternate connecting letters and numbers (e.g., 1-A-2-B …). Due to its increased complexity, it may be more clinically efficient in screening for cognitive impairment than Part A. While most people can complete Part B in 75 seconds, if someone requires more than 273 seconds to complete, cognitive impairment is likely [34].

Depression is another condition that increases the risk of physical dysfunction and global decline in older adults [35]. The Geriatric Depression Scale (GDS) is a 30-item patient-reported questionnaire; however, a 15-item GDS-Short Form (GDS-SF) has been highly correlated (*r* = 0.89) with the original GDS, therefore, the GDS-SF is likely more clinically efficient and applicable to physiotherapist practice [36]. The Patient Health Questionnaire (PHQ)-9 can also be used to screen for depression in older adults and consists of a 9-item questionnaire. A score of 4 has been established as a cutoff for possible depressive disorder. The test has demonstrated good sensitivity and excellent specificity [37].

### Healthy behaviors and readiness for change

Health habits and lifestyle choices play a substantial role in active aging as well as avoiding development of comorbid diseases or reducing their impact. The Physical Therapy Healthy Lifestyle Appraisal by Ingman et al. was designed to quantify current health behaviors as well as readiness for positive change [38]. (Table 3). It assesses the domains of healthy eating, physical activity (aerobic), sleep, stress management, and tobacco use. The person selects a statement that describes their typical behavior on each of the six items which are correlated with the stages of behavior change consistent with the transtheoretical model of health behavior change [39]. After this, the person then rates the importance and their confidence related to each of the areas on a 0 (not very important/not at all confident) to 10 (very important/very confident) scale. One benefit of this model is that the physiotherapist can establish and utilize a directed approach unique to each of the health behaviors.

**Table 3:** Physical Therapy Healthy Lifestyle Appraisal

Select the one statement that describes your …		Indicate how IMPORTANT it is today …(0 = not very important10 = very important)	Indicate how CONFIDENT you are today …(0 = I am not at all confident10 = I am very confident)
Healthy Eating	•I am following a healthy eating pattern.•I am thinking about or have recently started to follow a healthy eating pattern.•I have no intention of following a healthy eating pattern.	… for you to have a healthy eating pattern.	… that you can have a healthy eating pattern.
Physical Activity (Aerobic)	•I am physically active (aerobic). •I am thinking about or have recently become physically active (aerobic). •I have no intention of becoming physically active (aerobic).	… for you to be physically active (aerobic).	… that you can be physically active (aerobic).
Sleep	•I engage in healthy sleep behaviors. •I am thinking about or have recently started to engage in healthy sleep behaviors. •I have no intention of engaging in healthy sleep behavior.	… for you to engage in healthy sleep behaviors.	… that you can engage in healthy sleep behaviors.
Strengthening	•I engage in strengthening activity. •I am thinking about or have recently become engaged in strengthening activity. •I have no intention of engaging in strengthening activity	… for you to be engaged in strengthening activity.	… that you can be engaged in strengthening activity.
Stress Management	•I engage in behaviors to manage my stress. •I am thinking about or have recently started to engage in behaviors to manage my stress. •I have no intention of engaging in stress management behaviors.	… for you to engage in stress management behaviors.	… that you are able to engage in stress management behaviors.
Tobacco Use	•I do not currently use tobacco. •I am thinking about or have recently quit using tobacco. •I have no intention to quit using tobacco.	… for you to not use tobacco	that you can refrain from using tobacco.

Abridged from Ingman et al. [38]

### Frailty

Frailty is described as having a decreased physiologic reserve and an increased vulnerability to disease or death [40]. One of the earliest and most common frailty scales is the Fried Frailty Index which is widely cited in the literature [41]. There are five criterion that are evaluated and if a person is positive on three or more of the five indicators, they are considered frail thereby having an increased risk of death, disability, or institutionalization. These criteria include 1) self-selected gait speed over 4.6 m (15 ft), 2) frequency and duration of regular physical activity, 3) handgrip strength (via a handheld dynamometer), 4) self-perceived feeling of exhaustion, 5) and unintentional weight loss of more than 4.5 kgs (10 lbs.) in the last year [41]. In addition, the Rockwood Clinical Frailty Scale is another tool useful in subjectively categorizing a person based on their functional abilities from 1 (very fit) to 9 (terminally ill) [42, 43].

### Functional outcome, balance, and fall measures

The Short Physical Performance Battery (SPPB) is an objective physical assessment with significant predictive validity for hospitalization or death [44]. The SPPB consists of a series of three progressively harder static standing balance positions held for 10 seconds each (feet together, semi-tandem, tandem stance), self-selected gait speed assessment over 3 or 4 m (2 trials), and a 5-Times Sit to Stand test. Each of the three assessments is rated on a 4-point scale with a maximum score of 12 (highest function) and lowest score of 0.

The US’ CDC endorses several similar physical measures in the STEADI program including the Timed Up and Go (TUG) [45], the 30-Second Chair Stand test [46], and the Four Stage Balance Test [47]. It should be noted that the Four Test Balance Scale included in the SPPB is scored slightly different than the Four Stage Balance Test, although the testing positions are the same. An astute therapist may be able to strategically collect data in a time efficient manner to calculate the SPPB for its predictive validity purposes while also completing the CDC’s recommend physical measures which are included in a thorough algorithm of interventions based on fall risk categories [48].

### Falls

Quantification of falls is encouraged via a standardized, generalizable series of questions. For example, the Outcome and Assessment Information Set-Version D (OASIS-D) utilized in home healthcare within the US standardizes this line of inquiry by quantifying the outcomes of any falls within the last year. Each individual fall reported is categorized: No injury, minor injury (e.g., skin tears, abrasions, lacerations, superficial bruises, hematomas, sprains, or injury that causes pain), or major injury (bone fractures, joint dislocations, closed head injuries with altered consciousness, subdural hematoma) [49]. Although not explicitly included in the OASIS-D questions, a crucial line of inquiry is the mechanism of falls, therefore the physiotherapist is encouraged to prompt individuals for subjective details related to any falls that have occurred. Especially relevant are multiple falls that had a similar mechanism.

### Falls efficacy

The constructs of falls efficacy and fear of falling are closely related to frequency and severity of falls [50]. The STEADI program suggests the question “Do you feel unsteady when standing or walking?” to assess for fear of falling or fall efficacy as a screening question [28]. Two of the most used measures are the Falls Efficacy Scale International (FES-I) [51] and the Modified Falls Efficacy Scale (MFES) [52]. Both scales are self-reported questionnaires. The FES-I requires the individual to rate each of the 16 items from a 1 (not at all concerned) to 4 (very concerned) regarding performance on various functional tasks. There is a FES-I short form which has 7 questions instead of 16, and both scales have been translated into many languages. For the MFES, the person rates each of 14 functional tasks from a 0 (not confident) to 10 (completely confident). All three scales have demonstrated adequate validity and reliability [51–53].

### Home safety

Although home safety is frequently cited as an area to assess and modify with the aim of optimizing safe independent living, there is a notable lack of valid, reliable, objective measures of home safety risk [54]. One widely available screening checklist is the Home Falls and Accident Tool (Home FAST) [55]. There are two versions of the Home FAST, one designed for health professionals to administer (Home FAST-HP) and one designed for older adults to self-report their home assessment findings (Home Fast-SR). The Home FAST-HP consists of 25 items and the presence of 9 or more identified hazards on the Home FAST-HP scale is indicative of a higher risk of falling [56]. The Home FAST-SR consists of 88 items derived from the 25 items in the Home FAST-HP [57]. As the Home FAST-SR does not require a clinician to administer, it may be more clinically efficient during an already busy assessment session. However, the agreement between the Home FAST-SR and -HP was found to be good or excellent on only 52% of the assessment items [57]. It should be noted that much of the discrepancy in agreement between the two versions was because older women self-identified more home hazards than occupational therapists (OTs); however, it could not be confirmed whether the discrepancy was related to older adults over-estimating or the OT under-estimating the number of home hazards [57].

## Care delivery models

The results of a CGA should inform clinical decision making toward a multimodal prevention plan of care for the older adult population that addresses physical, medical, and social determinants of health. There are several comprehensive care delivery models available to optimize SAIP and active aging. The Community Aging in Place, Advancing Better Living for Elders (CAPABLE) program includes 10 visits over 4 months delivered in an older adult’s home with the intent to reduce personal and environmental risk factors to SAIP. The CAPABLE program utilizes a multidisciplinary approach of an OT, a registered nurse, and a handy worker providing visits in the home over 4–5 months to implement medical equipment and home modifications to assist with performance of activities of daily living as well as health behavior change and exercise to improve function and safety. CAPABLE has evidence for a 6-times return on investment when addressing both function and cost [58, 59]. Furthermore, a community-based Australian program, “Stay on your Feet,” is an informational initiative that involves “awareness raising, community education, policy development, engaging health professionals and interventions directly targeting individuals” [60]. It addressed risk such as “footwear/foot-care, vision, physical activity, balance and gait, medication use, and home and public environmental hazards” [60]. It was demonstrated that the Stay on your Feet program was cost effective by using a comprehensive approach which includes multimodal interventions that address physical domains and the built environment [61]. A notable component of this program is management of chronic conditions as well as, home and public environmental hazards and includes a widespread community awareness campaign [62]. Key outcomes included a 20% reduction in hospitalizations and a 22% reduction in self-reported falls [62]. Finally, the Home-based Older Persons Upstreaming Prevention Physical Therapy (HOP-UP-PT) Program has evidence of an 8-fold reduction in falls among older adults identified with a fall risk and will be described in more detail in the next section of this Masterclass [63]. The core HOP-UP-PT program entails 6 visits in the older adult’s home and three telehealth visits over a 7-month timeframe delivered by a physiotherapist. A key difference between the HOP-UP-PT program and CAPABLE and Stay on Your Feet programs is that the HOP-UP-PT program utilizes community-based referrals as a point of entry into the program and it has a substantial emphasis on balance exercises, physical activity, and leveraging health technology.

## HOP-UP-PT as a case example

As an example of how assessment and intervention can leverage the current science supporting SAIP and fall risk reduction in physiotherapist practice, HOP-UP-PT is one approach framed in the screening, assessments, and interventions mentioned in this Masterclass with strong scientific grounding (www.hopuppt.com). The HOP-UP-PT program was designed with an intent to combine efficient and effective tools and strategies to reduce falls and empower older adults to successfully age-in-place [64]. The program is novel in that it bridges public health and medical approaches to reducing falls and fall risks in older adults. Specifically, community-based senior centers and organizations that interact with older adults in the places they live and dwell are well positioned to recognize declines among their older adult residents. If these individuals observe functional deterioration or hear about a recent fall, then a referral to the HOP-UP-PT program can be facilitated, which offers a novel entry point into the healthcare system. In 2019, World Physiotherapy advocated for policy change to allow direct access to physiotherapy services [65]. This positions physiotherapists, who are trained in both prevention and rehabilitation, to straddle public health and clinically-based care delivery paradigms and to serve as a catalyst for innovative approaches to reducing falls.

The HOP-UP-PT program uses physical (e.g., TUG, SPPB), health (e.g., FCI, BP, PHQ-9, TMT-Part B, Physical Therapy Healthy Lifestyle Appraisal), fall risk (e.g., STEADI, MFES), and environmental (e.g., Home FAST-HP) assessments to guide person-centered interventions (e.g., Otago Exercise Program, motivational interviewing, home modification recommendation, self-BP monitoring, and wearable activity technologies). Improvements in the aforementioned domains were identified by experimental and observational studies of the HOP-UP-PT programmatic delivery, including a notable 8-fold reduction in falls among those at moderate/high risk of falls [63, 64, 66]. Additionally, investigation of long-term outcomes demonstrated trends toward sustained improvements in health outcomes, fall reduction, and positive perceptions of the HOP-UP-PT program interventions [67]. Each of the main interventions of the HOP-UP-PT program will now be described in more detail.

### Otago exercise program

Exercise and balance training are two well-known interventions that can improve fall risk and improve the likelihood of safe aging in place [68]. Targeted population-based interventions aimed at older adults, including Matter of Balance or the Otago Exercise Program (OEP) have evidence of their efficacy in reducing fall rates [69–71]. The OEP is one of the best-known evidence-based fall prevention programs and is intended for community-dwelling older adults and those in assisted living facilities [72, 73]. For these reasons, the OEP is the core exercise component of HOP-UP-PT. In a meta-analysis by Chiu et al., significant improvements were found in dynamic, static, proactive, and perceived balance and the greatest improvements were seen in older adults who performed the OEP in sessions longer than 30 minutes per day and in a group setting [74]. The OEP utilizes a standardized assessment to determine Otago exercise levels ranging from Level A (lowest exercise challenge) to Level D (highest exercise challenge) that are most appropriate to the older adult’s current functional level. The OEP guides the physiotherapist toward prescribing a safe and appropriate standardized exercise intervention. Safety when performing the exercises independently and unsupervised should be a primary consideration when prescribing the exercise level. To address aerobic fitness, walking is encouraged within the OEP. Participants should try to increase their time and/or distance each time they walk with the goal of sustained walking for 30 minutes. Providing a written handout with exercise instruction and an exercise tracking log is recommended.

### Motivational interviewing

We included motivational interviewing (MI) as part of HOP-UP-PT because it is a counseling technique that has been used and researched widely for more than three decades [75]. Its original intent was for use in substance abuse [76], but it has now been widely used to address behavior change in a variety of chronic diseases. Pignataro and Huddleston support MI as an approach to explore ambivalence and suggest that physiotherapists are ideally positioned to intrinsically motivate individuals to change unbeneficial health behavior [77]. MI has been described as a brief communication exchange with the aim of increasing motivation for change and then consolidating the commitment to change [78]. Arkkukangas et al. found that adherence to the OEP at 1-year follow up was significantly enhanced when delivered in conjunction with MI techniques [79]. Table 4 suggests a step-by-step approach to delivery of MI. This technique may be a useful adjunct to encouraging an older adult to articulate their perceived benefits of a given behavior change. This will help to express ways in which the change will positively impact their life and successfully age-in-place, as well as aid in resolving issues of non-action towards positive health behaviors.

**Table 4:** 10-step Process of Motivational Interviewing

Step	Goal of the Step	Physiotherapist action steps
1	Initiation of the subject	Use open ended questions to begin conversation about the health behavior
2	Explore their reasoning	Ask about reasons behind current behavior
3	Reflective listening	Provide brief summary statements to reflect back to them the thoughts and feelings being expressed about their reasons behind their current behavior
4	Explore benefit of behavior change	Use open ended questions to get them to articulate what the benefits of changing their behavior might be
5	Reflective listening	Provide brief summary statements to reflect back to them the thoughts and feelings being expressed about their reasons behind their current behavior
6	Explore ambivalence	Using a 0–10 scale (0 = not important at all, 10 = essential), have them rate how important it is to them to make this change
7	Explore ambivalence	Ask them why they rated it at that number and not a lower number
8	Explore and support their self-efficacy	Ask them on a scale of 0–10 (0 = not confident at all, 10 = extremely confident) how confident they are that they can change Ask them why they rated it at that number and not a lower number
9	Explore and support their self-efficacy	Reinforce if they have had some success in the past and willingness to even consider and discuss changing the behavior
10	Explore their future plans and ideas	Encourage the patient to come up with solutions for themselves; Refrain from trying to suggest solutions

### Wearable Activity Monitor

Use of a wearable activity monitor is recommended in the older adult population to promote exercise program compliance through the provision of feedback and extrinsic motivation [80]. In a systematic review, Cooper et al. found that accelerometer use significantly increased physical activity levels in older adults [81]. Collecting activity data from the device can provide helpful insight to a physiotherapist on participant activity volumes, but it is not absolutely necessary for successful risk reduction.

### Home blood pressure monitoring

Studies have identified that 75% of home health care patients and 62% of outpatients under the care of physiotherapists were identified as having either pre-hypertensive or hypertensive blood pressure measures [21, 23]. Therefore, availability of an automatic blood pressure unit in the home would offer another level of screening in that the older adult could be trained to self-identify factors which may predispose them to a fall (e.g., orthostatic hypotension) as well as hypertensive measures which may warrant further workup by a medical professional. This piece of equipment is simple to learn and use and has potentially lifesaving benefits in the early identification of potential cardiovascular issues. Recording BP measures regularly is recommended to have documentation of blood pressure patterns over time useful in communicating irregularities with interdisciplinary colleagues [20].

### Home modification recommendations

There is an increasing body of evidence that SAIP is closely correlated with a safe and accessible home environment, not only to prevent falls but to also optimize community accessibility [82]. The previously mentioned Home FAST assessment is a useful environmental-focused fall screening tool and results can provide direction for home modifications [83]. Removing electrical cords and/or other clutter that may impede the ability to walk safely through the home, recommending the repair of flooring in poor condition, and suggesting loose floor mats be secured with double sided tape are some examples of home modification recommendations. More extensive issues within the built environment can be addressed with the assistance of a local community center. Community centers serving older adults may have provisions for minor home repair grants, local handy worker services, or pro bono home modifications.

### Community reintegration

Many locales have community-facing organizations that serve senior citizens by providing valuable resources to support positive behavior change and facilitate social support systems [64]. Martín-Borràs et al. found that referrals to community services and exercises that included social support and community integration improved long term physical activity levels in older adults [84]. As the services that these organizations offer are not routinely integrated within the medical model, physiotherapists must proactively reach out to develop partnerships to facilitate utilization of resources for safe aging. Beneficial offerings may include group exercise programs, book clubs, meal delivery services, transportation, and recreational activities.

### Referrals

Physiotherapists can leverage their integration within the medical community by providing referrals to other healthcare team members for any identified evolving health or cognitive issues. Boissonnault and Ross identified 78 case reports where physiotherapists identified issues that required physician assessment and referrals were made to optimize outcomes and ensure safety [85]. Referrals to outpatient physiotherapy or other rehabilitation providers should also be considered when a need is identified.

## Conclusion

There is a substantial body of evidence for screening, assessment, and individualized interventions to promote fall reduction and SAIP. Assessments and interventions span multiple domains that address physical health, environmental, and social determinants of successful and active aging. Programs integrating both community and healthcare approaches have the strongest evidence for their utility; however, implementation of these preventative approaches are lagging behind the substantial population growth of those over 65 years [86]. Embracing this framework is essential to improving the value of healthcare delivery for both older adults and the care providers.

## Data Availability

All data generated or analyzed during this study are included in this published article.

## Abbreviations

AGS: American Geriatrics Society
BMI: Body mass index
BP: Blood pressure
CAPABLE: Community Aging in Place, Advancing Better Living for Elders
CCI: Charlson Comorbidity Index
CDC: Center for Disease Control and Prevention
CGA: Comprehensive geriatric assessment
FCI: Functional Comorbidity Index
FES-I: Falls Efficacy Scale International
GDS: Geriatric Depression Scale
GDS-SF: Geriatric Depression Scale-Short Form
Home FAST: Home Falls and Accident Tool
HOP-UP-PT: Home-based Older Persons Upstreaming Prevention Physical Therapy
HTN: Hypertension
MI: Motivational interviewing
Min(s): Minutes
MFES: Modified Falls Efficacy Scale
MMSE: Mini Mental State Exam
OASIS-D: Outcome and Assessment Information Set-Version D
OEP: Otago Exercise Program
PHQ-9: Patient Health Questionnaire 9
SAIP: Safe aging in place
SPPB: Short Physical Performance Battery
STEADI: Stopping Elderly Accidental Death and Injury
TMT: Trail Making Test
TUG: Timed-Up-and-Go
USD: United States Dollars
